# Domain I of β2GPI is capable of blocking serum IgA antiphospholipid antibodies binding in vitro: an effect enhanced by PEGylation

**DOI:** 10.1177/0961203319851571

**Published:** 2019-05-24

**Authors:** A Albay, B Artim-Esen, C Pericleous, C Wincup, I Giles, A Rahman, T McDonnell

**Affiliations:** 1 Centre for Rheumatology Research, Division of Medicine, University College London, Department of Medicine, Rayne Institute, London, UK; 2 Istanbul University, Istanbul Faculty of Medicine, Department of Internal Medicine, Division of Rheumatology, Turkey; 3 Imperial College London, Imperial College Vascular Sciences, National Heart & Lung Institute, ICTEM, London, UK

**Keywords:** Antiphospholipid syndrome, Domain I, PEGylation

## Abstract

**Objectives:**

This study aims to inhibit antiphospholipid syndrome (APS) serum derived IgA anti-beta-2-glycoprotein I (aβ2GPI) binding using Domain I (DI).

**Methods:**

Serum from 13 APS patients was tested for IgA aβ2GPI and Anti-Domain I. Whole IgA was purified by peptide M affinity chromatography from positive serum samples. Serum was tested for IgA aβ2GPI binding in the presence and absence of either DI or of two biochemically modified variants containing either 20 kDa of poly(ethylene glycol) (PEG) or 40 kDa of PEG.

**Results:**

Significant inhibition with DI was possible with average inhibition of 23% (*N* = 13). Further inhibitions using 20 kDa PEG-DI and 40 kDa PEG-DI variants showed significant inhibition (*p* = 0.0001) with both the 40 kDa PEG-DI and 20 kDa PEG-DI variants showing increased inhibition compared with DI alone (*p* = 0.0001 and *p* = 0.001, *n* = 10).

**Conclusions:**

Inhibition of IgA aβ2GPI by DI is possible and can be enhanced by biochemical modification in a subset of patients.

## Introduction

Antiphospholipid syndrome (APS) is an autoimmune disorder defined by both laboratory criteria and clinical presentation. Three serological criteria for APS are included: IgG and IgM anti-cardiolipin (aCL) and anti-beta-2-glycoprotein I (aβ2GPI) ELISAs, and the lupus anticoagulant test. Whilst IgG and IgM antiphospholipid antibodies (aPL) are well recognized, the importance of IgA aPL is still controversial.^1,2^

Diagnostically IgA aPL has been evaluated in several studies and found even in patients negative for the classical diagnostic antibodies^2–4^ but who have typical clinical features of APS (seronegative APS). Part of the controversy regarding IgA aCL and aβ2GPI is due to the variable prevalence reported in cohorts.^5^ Some groups found significant clinical associations with thrombosis and APS diagnosis,^2,6^ whilst others did not.^7^

In vitro studies of IgA aPL have shown pathogenic potential in a mouse model of APS^8^ implying potential pathogenicity in patients in the absence of IgG and IgM aPL antibodies. Other studies have suggested this pathogenicity has already been seen in patients.^2,9,10^

The standard of treatment for APS patients is lifelong anticoagulation either by warfarin, heparin or, more recently, using direct oral anticoagulants such as rivaroxaban,^11^ though these have been shown to have their own risks.^12^ This has led to a spate of research into novel therapeutics for APS.^13–15^

These novel therapeutics target β2GPI rather than the clotting cascade, and the majority of research has been carried out on the IgG subclass. This study used one of these novel therapeutics (Domain I) and its modified forms (PEGylated DI) to test whether it was possible to disrupt the binding of patient IgA antibodies to β2GPI immobilized in an ELISA.

## Materials and methods

### Patient selection

Thirteen patients were selected from the University College London Hospital (UCLH) and University of Istanbul cohorts of patients with APS, with or without systemic lupus erythematosus (SLE). Patient sera were tested previously for IgG, IgM and IgA aβ2GPI and Anti-Domain I positivity.^2^ Data on positivity for aCL and lupus anticoagulant (LA) were taken from clinic records. Patients with known IgA positivity were selected for this study.

### Preparation of DI and PEG-DI

DI was prepared as previously and PEGylated as described.^13^ Briefly: protein was produced bacterially in *Escherichia Coli* and folded manually through dilution. Purification was by Immobilised Metal Affinity Chromatography (IMAC), Cation Exchange Chromatography (CEX) and gel filtration. PEGylation was carried out using TheraPEG^R^ targeting disulphide bonds. Two conjugates were produced using 20 kDa and 40 kDa poly(ethylene glycol) (PEG). Expression tag was removed by FXa cleavage and protein was dialysed against phosphate-buffered saline before quantification and storage. Concentrations of PEGylated conjugates were expressed in terms of mass of DI.

### Serum IgG depletion

Serum samples of IgG/IgA aβ2GPI positive patients were depleted using protein G beads. Serum samples were diluted 1:1 with binding buffer (10 mM NaPO4, 100 mM NaCl, pH 7.4) and applied to 100 µl of protein G beads (Sigma) for 1 h rotating at room temperature. Samples were centrifuged (13,000 *g*, 10 min, 4℃) and supernatant stored for analysis. Samples were washed with 500 µl of binding buffer, centrifuged and bound IgG was eluted with glycine (0.1 M, pH 2.3) rotating for 1 h before centrifugation and neutralization with 10 µl of Tris (1 M, pH 9.0) per 100 µl of glycine.

### Serum IgA purification

IgA antibodies were purified using a peptide M column (Sigma). Purification was as for serum IgG depletion with the following exception: serum was diluted 2:1 with 10 mM sodium phosphate pH 7.2 and loaded onto a 1 ml column.

### Direct IgA aβ2GPI ELISA and inhibition ELISA

Direct ELISAs for IgG and IgA aβ2GPI were as previously described.^2^ The IgA aβ2GPI inhibition ELISA was carried out as in McDonnell et al.^13^ with the following alterations: secondary antibody was an anti-human IgA (Abcam) at a dilution of 1:10,000 and substrate was applied for 20 min before being stopped. DI was used at concentrations ranging from 25 µg/ml to 100 µg/ml, 20 kDa and 40 kDa PEGylated proteins were used at 50 µg/ml. Purified IgA was used between 12.5 µg/ml and 25 µg/ml of IgA, depleted serum was used at dilutions between 1:12.5 and 1:25 to achieve an optical density (OD) of between 0.4 and 0.8.

## Statistical analysis

PRISM and Stata programmes were used to carry out one-way analysis of variance and non-parametric Mann–Whitney *t*-tests.

## Results

Table 1 shows the characteristics of the 13 patients tested; 11 had venous thrombosis, one had suffered pregnancy morbidity and one patient had both venous thrombosis and pregnancy morbidity. The mean IgA aDI and aβ2GPI levels were high (42.4 and 68.7 units respectively). Lower levels of IgG aDI and aβ2GPI were seen in these patients (22.6 and 26.1 units respectively). Of the 13 patients, seven also had SLE and 10 had LA positivity. Patients were recruited from both the UCLH cohort of APS patients and a Turkish cohort from the University of Istanbul.

**Table 1:** Demographic and disease based details for patients involved in the study

	APS *N* = 13
VT	11
PM	1
VT/PM	1
Gender	Four male, nine female
Age	46.2 (±19.1)
IgA aDI	38 (±32)
IgA aβ2GPI	65 (±39)
IgG aDI	21 (±35)
IgG aβ2GPI	25 (±34)
LA	10
SLE	7

a β2GPI: anti-beta-2-glycoprotein I; aDI: anti-Domain I; APS: antiphospholipid syndrome; LA: lupus anticoagulant; PM: Pregnancy Morbidity; SLE: systemic lupus erythematosus; VT: Venous Thrombosis.

### Domain I inhibits patient IgA binding to β2GPI in serum in a dose-dependent manner

**Figure 1:**
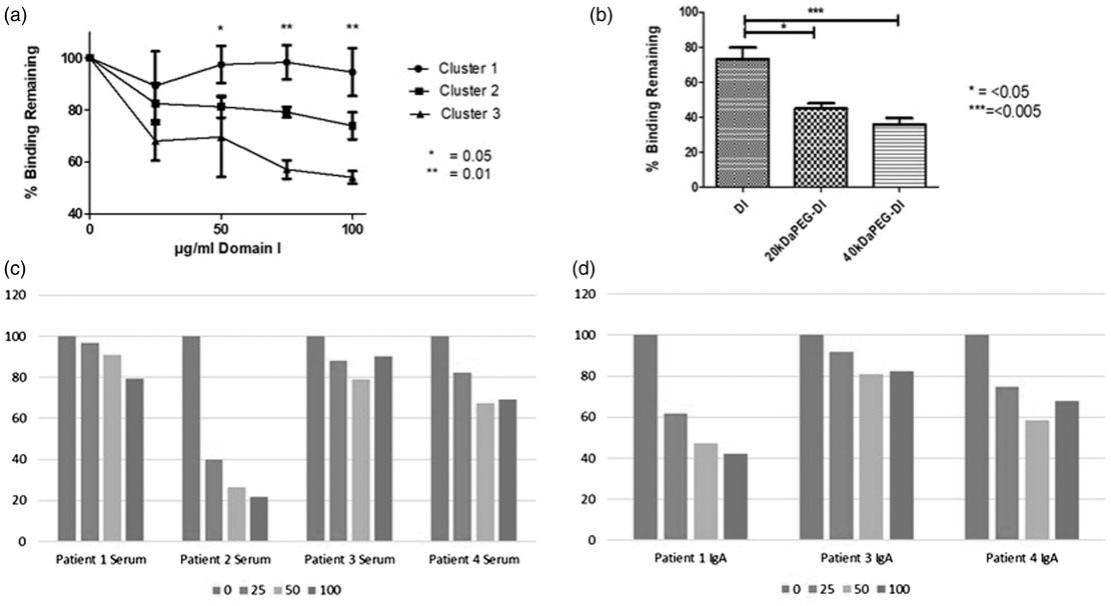
Results of assays to measure inhibition of IgA binding to β2GPI. (a) The inhibition of IgA aβ2GPI in serum from 13 patients separated into three clusters. Cluster 1 had no inhibition, cluster 2 had moderate inhibition and cluster 3 had the highest inhibition. Significant differences are seen between clusters 1 and 3 at concentrations ≥50 µg/ml DI and between clusters 1 and 2 at ≥75 µg/ml DI. (b) Inhibition by DI and PEG-DI in 10 of these patients tested with 20 kDa PEG-DI and 40 kDa PEG-DI. Both PEGylated variants show significantly enhanced inhibition. (c) Inhibition by non-PEGylated DI in IgG depleted serum for all four patients from cluster 3. Inhibition can be seen in all four patients and is dose-dependent in three cases. (d) Inhibition of purified IgA from three of the patients from cluster 4 (it was not possible to purify sufficient IgA from serum of patient 2). Inhibition can be seen in all three patients. aβ2GPI: anti-beta-2-glycoprotein I; DI: Domain I; PEG: poly(ethylene glycol)

Initially serum samples from patients (*N* = 13) were screened for inhibition with increasing doses of DI (0–100 µg/ml). As shown in Figure 1(a) patients clustered into three groups: no or little inhibition (cluster 1, *n* = 4), low inhibition (<40% inhibition at 100 µg/ml, cluster 2, *n* = 5) and high inhibition (≥40%inhibition at 100 µg/ml, cluster 3, *n* = 4). Dose-dependent inhibition for clusters 2 and 3 can be seen in Figure 1(a). Significant differences were seen between clusters 1 and 2 and clusters 1 and 3 (Figure 1(a)).

Inhibition with 20 kDa PEG-DI and 40 kDa PEG-DI was carried out at 50 µg/ml. Inhibition can be seen in Figure 1(b), which includes 10 patients from all three clusters. There was significant (*p* < 0.05) enhancement of inhibition for both PEGylated forms in comparison with non-PEGylated DI.

Another interesting result was the difference in inhibition for patients with high and low IgG and IgA. Patients in cluster 1 with low inhibition also had the lowest levels of both IgG and IgA aβ2GPI (5.08 GBIU and 44.48 ABIU) and aDI (6.02 GDU, 19.92 aDU). Those patients with moderate inhibition in cluster 2 had high IgA (77.08 ABIU, 45.21 ADU) but lower IgG (18.8 GBIU, 9.82 GDU) whereas those in cluster 3 with the highest inhibition had both high IgG and IgA aβ2GPI (64.28 ABIU, 56.63 GBIU) and aDI (53.65 ADU and 52.08 GDU).

To analyse further the high-level inhibition of IgA aβ2GPI binding in serum from patients in cluster 3 we carried out further tests on serum depleted of IgG and on purified IgA samples.

### IgG depleted serum retains its IgA aβ2GPI activity, which is inhibited by non-PEG-DI

Samples from three of the four cluster 3 patients (patients 1, 2 and 4 in Figure 1(c)) showed significant IgG aβ2GPI depletion demonstrated by loss of >80% binding in an IgG aβ2GPI ELISA (Supplementary Material Figure 1 online). IgG-depleted samples retained their IgA aβ2GPI activity sufficiently to carry out further inhibition experiments using a dilution that gave an OD between 0.4 and 0.6 (12.5–25 µg/ml) in the absence of inhibitor. Doses of DI ranging between 25 µg/ml and 100 µg/ml showed dose-dependent inhibition of IgA aβ2GPI activity (Figure 1(c)) for patients 1, 2 and 4.

### Inhibition of purified IgA aβ2GPI binding

Purified IgA was derived from serum of three patients (1, 3 and 4). Two of these (1 and 4) showed dose-dependent inhibition between 25 and 100 µg/ml of DI. This can be seen in Figure 1(d). These results mirrored the effects of non-PEGylated DI in IgG depleted serum.

## Discussion and conclusions

In this study we have shown that it is possible to inhibit the binding of both purified and serum IgA from APS patients from binding to β2GPI.

Interestingly, the inhibitory effect seen with DI was enhanced by PEGylation. This is an unusual phenomenon; the majority of PEGylated proteins lose their natural affinity for ligands. One possible explanation is that this apparent increase may also be due to stabilizing the epitope on DI or due to an increased purity of DI. An alternative theory may be that DI has aggregated and as such is presenting multiple epitopes. However, we studied this possibility via a gel-filtration method and also with native polyacrylamide gel electrophoresis and saw no aggregation. The potential for aggregation is also higher in the non-PEGylated protein as PEGylation has been shown to reduce aggregation.^16,17^ These results are in contrast to our previous research using IgG aβ2GPI antibodies where significant inhibitory potential of DI was retained but not enhanced after PEGylation.^13^

The strongest inhibition was seen in those patients with both high IgA aDI/aβ2GPI and high IgG aDI/aβ2GPI. To distinguish the effects of IgG and IgA inhibition we carried out experiments using IgG-depleted serum and purified IgA. The results obtained with total serum, IgG-depleted serum and purified IgA cannot be compared directly in a quantitative way due to the differences in sample preparation. However, a qualitative comparison shows that inhibition of IgA aβ2GPI was seen in IgG depleted samples and in purified IgA for all but one sample. This sample (from patient 3) was notable because it had particularly high levels of IgG and IgA aβ2GPI binding and because it was not possible to deplete the IgG aβ2GPI. It is possible that the poor inhibition in this sample was because of a particularly high level of antibody binding (both isotypes).

This study utilized patients from two different centres with different ethnic backgrounds; however, the numbers are small. Further studies with larger cohorts would aid in interpreting these results.

This study shows it is possible to inhibit the binding of IgA from patients to β2GPI in an ELISA using DI and PEG-DI and suggests that the most efficient inhibition is seen in patients with dual positivity for IgG and IgA antibodies. It also raises the interesting result that PEGylation may increase the ability to inhibit IgA from patients; however, larger studies are required for this to be confirmed.

## Key messages

- Domain I of beta-2-glycoprotein I is capable of inhibiting IgA antibody binding.
- Biochemical modification of Domain I can increase antibody binding.
- Patients’ serum most inhibited by Domain I contained both high IgA and IgG.
